# Significantly enhanced dielectric constant and energy storage properties in polyimide/reduced BaTiO_3_ composite films with excellent thermal stability

**DOI:** 10.1039/c8ra10434d

**Published:** 2019-03-07

**Authors:** Shuangshuang Yue, Baoquan Wan, Yunying Liu, Qiwei Zhang

**Affiliations:** Inner Mongolia Key Laboratory of Ferroelectric-related New Energy Materials and Devices, Inner Mongolia University of Science and Technology 7# Arding Street, Kun District Baotou 014010 China zqw8000@imust.edu.cn; School of Chemistry and Chemical Engineering, Inner Mongolia University of Science and Technology 7# Arding Street, Kun District Baotou 014010 China; Key Laboratory of Integrated Exploitation of Bayan Obo Multi-Metal Resources, Inner Mongolia University of Science and Technology Baotou 014010 China

## Abstract

In this work, reduced BaTiO_3_ (rBT) particles with a large number of defects sintered in a reducing atmosphere (95N_2_/5H_2_) were introduced into polyimide (PI) matrix without using any modifier or surfactant components. The rBT/PI composite films fabricated by an *in situ* polymerization method showed significantly enhanced dielectric constant and energy storage density. The dielectric constant of the rBT/PI composite with 30 wt% rBT reached up to 31.6, while maintaining lower loss (tg *δ* = 0.031@1000 kHz) compared to pure PI (*ε*_r_ = 4.1). Its energy storage density (9.7 J cm^−3^ at 2628 kV cm^−1^) was enhanced by more than 400% over that of pure PI (1.9 J cm^−3^ at 3251 kV cm^−1^), and was greater than the energy density of the best commercial biaxially-oriented-polypropylenes (BOPP) (1.2 J cm^−3^ at 6400 kV cm^−1^). The energy storage efficiency was around 90% due to the linear dielectric performance of rBT/PI composite films. The improved dielectric constant and energy storage density could be attributed to the combined effect of the interface interaction between two phases and the surface defects of rBT induced by the reducing atmosphere. Therefore, rBT/PI composite films with high dielectric constant, energy storage density and storage efficiency may have potential applications in the preparation of embedded capacitors.

## Abstract 2

The semiconductor properties of reduced barium titanate, with a high energy density of 9.7 J cm^−3^.

## Graphical abstract

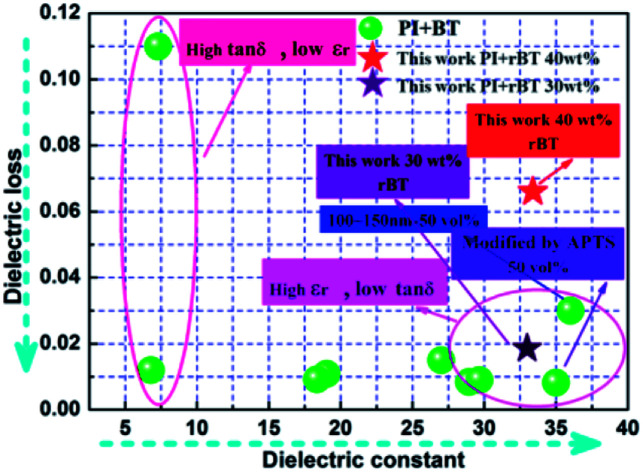

## 1 Introduction

Polymer materials with high dielectric constant play critical roles in the modern information and electronic industry as embedded capacitors and charge-storage devices because they are light weight, multifunction-integrated and miniaturization-friendly.^1–5^ Pure polymer materials have many merits including excellent flexibility, low-temperature processing, high electric breakdown strength, and so on.^6^ However, their low dielectric constant (typically less than 10) impedes their practical applications in capacitors.^7^ A promising strategy to enhance the dielectric constant of polymer materials is to incorporate ferroelectric materials with high dielectric constant (such as titanium dioxide (TiO_2_),^8,9^ BaTiO_3_,^10,11^ Pb(Zr,Ti)O_3_,^12,13^ BaSrTiO_3_,^14,15^ K_0.5_Na_0.5_NbO_3_ (ref. 16 and 17) and CaCu_3_Ti_4_O_12_ (ref. 18 and 19)) into a polymer matrix to form composites.^20–23^ This approach has been extensively studied for various polymer matrices including poly(vinylidene fluoride) (PVDF), polymethylmethacrylate (PMMA),^24^ polyimides^25^ and epoxy resins.^26^ The dielectric constant of ferroelectric/polymer composite system can be improved by a few times compared to the pure polymer when a high content of ferroelectric particles is incorporated (>50 vol%). Unfortunately, the breakdown strength and energy storage density of these composites are low.^27–29^ Therefore, it is still a challenge to find an effective way to achieve both high dielectric constant and breakdown strength.

**Fig. 1:**
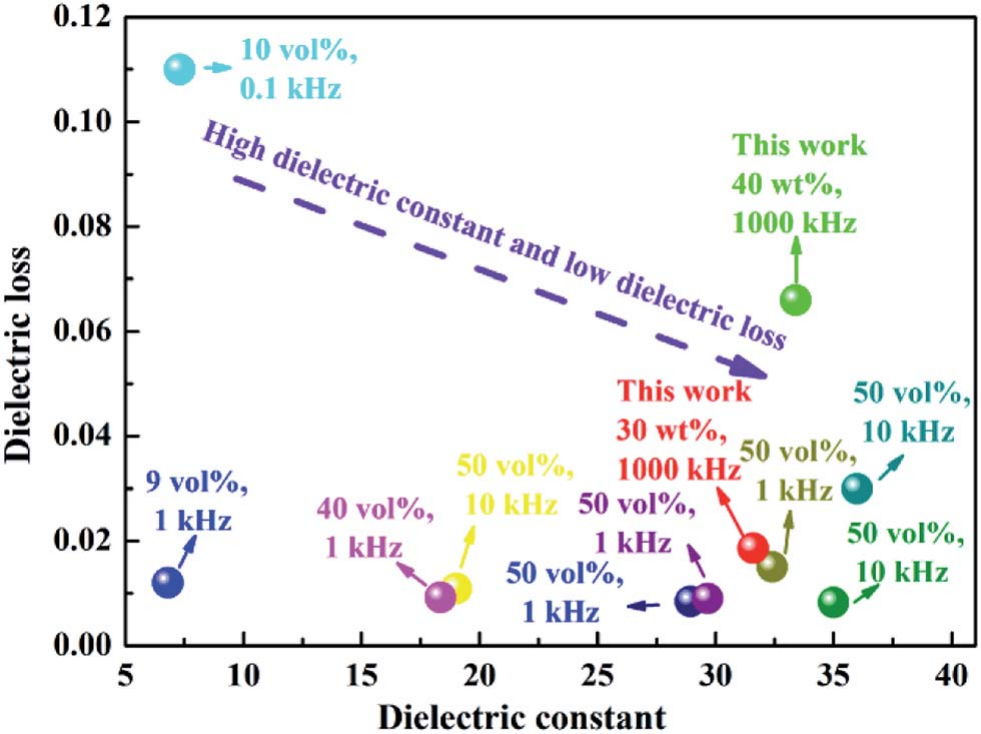
The dielectric constant and loss of BT/PI composite materials reported in literature.^36–43^

Among the many polymers, polyimides (PI) are widely used as packaging materials, insulating layers, circuit boards and interlayer dielectrics due to their high tensile strength, superior mechanical properties, high glass transition temperature, good resistance to solvents and excellent thermal stability.^30–33^ PI is now considered to be a promising candidate for polymer composite dielectrics with good temperature stability. For example, Li *et al.* reported that titanium oxide/PI composites exhibited dielectric constant (*ε*_r_) of 10.6 and low dielectric loss of <0.03 with 10 wt% high-aspect-ratio titanium oxide nanowires.^34^ The dielectric properties for some recently reported BT/PI composites are shown in Fig. 1. From these reported results, it can be seen that it is difficult to simultaneously achieve high dielectric constant and low loss (low dielectric breakdown strength) in PI-based composites. In addition to the dielectric properties at room temperature, it is necessary to systematically investigate the thermal stability of PI composites for dielectric capacitors in practical application.

Compared to BaTiO_3_ ceramics fabricated in air, BaTiO_3_ materials sintered in reducing atmosphere exhibit excellent dielectric properties due to their semiconducting properties.^35^ In this work, reduced BaTiO_3_ (rBT) particles were introduced into PI matrix to form rBT/PI composite films by *in situ* polymerization. Notably, the composites were obtained through simply mixing the precursor solution (PAA) and rBT suspension without using any modifier or surfactant components. The dielectric and energy storage properties of rBT/PI composite films were systematically studied. Significantly increased dielectric constant and energy storage density were realized in the as-prepared composite films.

## 2 Experiments

### 2.1. Materials

BaTiO_3_ (BT, 99.9%), BaCO_3_ (99.8%) and TiO_2_ (99.8%) were purchased from Alfa Aesar (China) Chemicals Co., Ltd. 4,4′-Diaminodiphenyl ether (ODA), pyromellitic anhydride (PMDA) and *N*,*N*-dimethylformamide (DMF, SP grade) were purchased from Aladdin, Shanghai, China. Absolute alcohol (AR grade) was purchased from Tianjin Reagents Co. Ltd. All the reagents were used as received without further purification. Deionized water was prepared in our laboratory.

### 2.2. Preparation of reduced BaTiO_3_ (rBT)

rBaTiO_3_ powder was prepared by a solid-state reaction described by the following equation:

1 BaCO_3_ + TiO_2_ → BaTiO_3_ + CO_2_

High purity metal oxides or carbonates (BaCO_3_, TiO_2_) were used as the raw materials in this work. These powders were weighed and ball-milled in a polyethylene bottle with ethyl alcohol and zirconia balls for 24 h, and then dried in an oven at 60 °C for 6 hours. The resulting powder was calcined at 1000 °C for 4 h in air. The calcined powder was ball-milled once in ethyl alcohol for 24 h, and then dried in an oven at 60 °C. Finally, the reduced BT powder was prepared by heat treatment at 1250 °C for 2 h in a reducing atmosphere of N_2_ and H_2_ (95/5).

### 2.3. Preparation of rBT/PI composite films

**Fig. 2:**
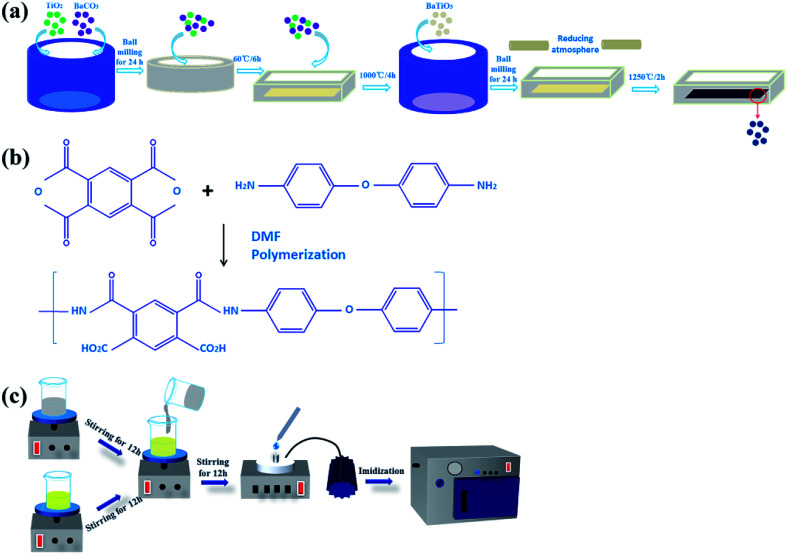
The preparation process of (a) rBT powder samples, (b) PAA solution (the precursor of PI), and (c) rBT/PI composite films.

The rBT/PI composites were prepared by *in situ* polymerization. First, ODA was dispersed into DMF solvent, and stirred until ODA was completely dissolved in DMF. Then, PMDA was added slowly to ensure complete dissolution, and stirred for 12 h. The as-prepared rBT powder was added into DMF solvent with the contents of 0 wt%, 5 wt%, 10 wt%, 20 wt%, 30 wt%, 40 wt% and 50 wt%, to form a series of suspensions. The suspensions were ultrasonicated for 1 h and then stirred for 12 h. Subsequently, the two solutions were mixed together, ultrasonicated for 1 h and then stirred it for 12 h. Finally, the rBT/PI composite films were prepared *via* solution casting method on the ITO substrate. The films were subsequently vacuum-dried at 80 °C for 2 h to volatilize the solvent, and then dried at 100 °C/2 h, 120 °C/2 h, 150 °C/1 h, 200 °C/1 h, 250 °C/1 h and 300 °C/1 h to convert completely into rBT/PI composite films. The detailed experimental procedure is described in Fig. 2.

### 2.4. Characterization of structure and dielectric properties

The surface and cross-section morphology of the samples were examined by field emission scanning electron microscope (FESEM; JEOL, JSM-6701F) equipped with energy dispersive spectroscopy (EDS, OXFORD). Phase structure of samples was analyzed by an X-ray diffractometer using Cu-Kα radiation (D8 Advanced, Bruker, Germany). Thermal gravimetric analyzer (TGA; TA, STA 449C) was used to perform the thermal analysis of composite films from room temperature to 800 °C with a heating rate of 10 °C min^−1^ under Ar flow. Bonding energy of elements in BT was measured using X-ray photoelectron spectroscopy (XPS; Kratos, Axis Supra) with Al Kα radiation (*hν* = 1486.6 eV). Both sides of composite films were sputtered with gold (1 mm diameter and 60 nm thick) as electrodes for measurement of electrical properties. A TH2828S LCR meter was used to measure dielectric properties of composites from frequency 0.1 kHz to 1000 kHz at room temperature. The electric breakdown strength (*E*_b_) was measured using a high-voltage tester (ET2671A, China) at room temperature. During breakdown strength measurements, at least 8 specimens were selected for calculating the average *E*_b_. The polarization–electric (*P*–*E*) field loops for rBT/PI composites were measured using a Premier II ferroelectric test system (Radiant Technologies, Inc. Albuquerque, NM).

## 3 Results and discussion

### 3.1. Structure characterization

**Fig. 3:**
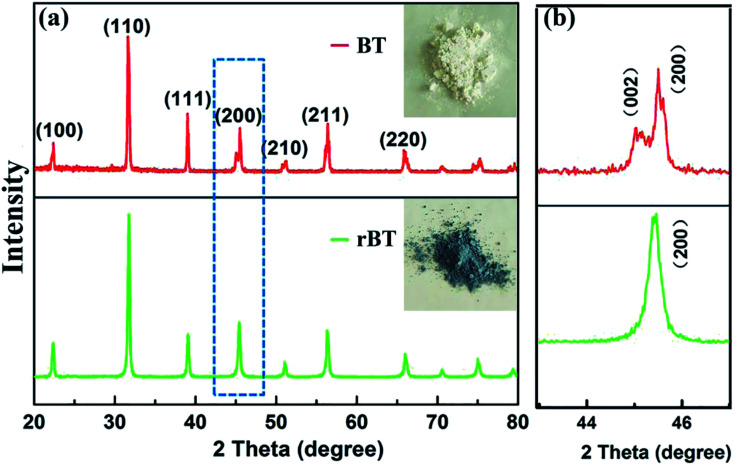
(a) XRD patterns of BT and rBT, the insets are photographs of BT and rBT powders, and (b) the refined peaks near 45 for BT and rBT.

X-ray diffraction (XRD) patterns of BT and rBT powders are shown in Fig. 3(a). The refined scanning (2*θ* = 40–50°) patterns are also shown in Fig. 3(b). The reduced BaTiO_3_ had similar XRD pattern as pure BaTiO_3_ phase. All samples belonged to typical perovskite structure, and no secondary phases were detected. The rBT exhibited a tendency to transform from tetragonal phase to cubic phase, which can be seen from the (002) and (200) peaks. These two peaks gradually overlapped at about 2*θ* ≈ 45°. Moreover, BT powder sintered under reducing atmosphere exhibited gray color while BT powder sintered in air was white, as shown in the inset of Fig. 3(a). According to many reported results, the blue color of rBaTiO_3_ powder was attributed to the presence of Ti^3+^ formed from Ti^4+^, which may be related to the following two mechanisms: (1) a direct donor-doping process: 2Ti^4+^ + H_2_ → 2H^+^ + 2Ti^3+^; (2) the loss of oxygen during heat treatment in an atmosphere with low oxygen partial pressure: O_2_^+^ → 1/2O_2_ + 2e^−^.^44–47^

**Fig. 4:**
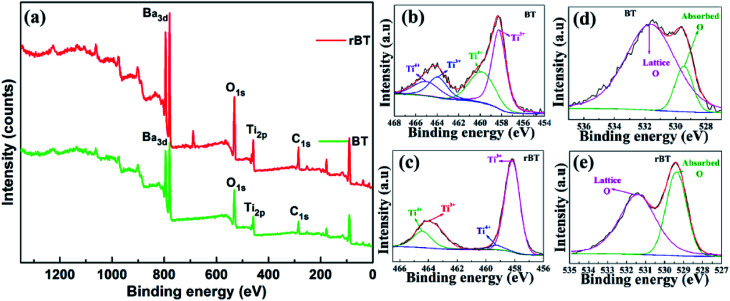
(a) XPS spectra of BaTiO_3_ and reduced BaTiO_3_ powders, (b) and (c) Ti_2p_ and (d), (e) O_1s_ XPS spectra of BaTiO_3_ and reduced BaTiO_3_, respectively.

The transition of Ti^4+^ to Ti^3+^ and the existence of oxygen vacancies can be further confirmed by XPS analysis, as shown in Fig. 4(b)–(e). The observed XPS spectra of BaTiO_3_ and rBaTiO_3_ were consistent with the reported data in literature.^48,49^ The Ti_2p_ peak of BaTiO_3_ was split into 4 peaks, which were located at 465.0 eV and 459.8 eV corresponding to Ti_2p3/2_ and Ti_2p3/2_ peaks of Ti^4+^, and 463.9 eV and 458.26 eV corresponding to Ti_2p1/2_ and Ti_2p1/2_ peaks of Ti^3+^, respectively.^50^ The Ti_2p_ peak of rBaTiO_3_ can also be deconvoluted into 4 peaks, two of which were located at 464.4 eV and 459.3 eV consistent with the Ti_2p3/2_ peaks of Ti^4+^, and the other two peaks were located at 463.5 eV and 458.1 eV, corresponding to the Ti_2p1/2_ peaks of Ti^3+^. The ratio of Ti^3+^/Ti^4+^ increased from 1.4 to 3.0. The O_1s_ signal displayed two components at 531.6/531.4 eV and 529.4/529.4 eV, which were assigned to lattice O and absorbed O, respectively. The ratio of lattice O/absorbed O decreased from 6.1 to 1.5. The surface area and ratios under the peaks of the BaTiO_3_ and rBaTiO_3_ ceramics are listed in Table 1. The area of Ti^3+^ and absorbed O of rBaTiO_3_ increased significantly compared to that of BaTiO_3_ (as shown in the Fig. 4(b)–(e) and Table 1), suggesting that most Ti^4+^ ions were reduced to Ti^3+^ ions and the oxygen vacancies increased under the reducing atmosphere (N_2_/H_2_).^51^ According to the XPS survey spectra of BT and rBT powders, the elemental concentration were determined by a Thermo Avantage software. The Ba, Ti, O, and C atomic percentages for BT and rBT powders (BT/rBT) are about 8.85%/7.65% (Ba_3d5_), 8.72%/8.33% (Ti_2p_), 45.54%/40.29% (O_1s_), and 36.89%/34.94% (C_1s_), respectively. It is found that Ba/Ti ratios is close to 1, which is in agreement with BaTiO_3_ molecular formula, whereas rBT show a difference of Ti/O ratios, suggesting that there exist many O vacancies.

**Table 1:** Fitting parameters of the Ti_2p_ and O_1s_ XPS spectra of rBaTiO_3_ and BaTiO_3_ powders

Peak	Position (eV)	Area	FWHM (eV)
Ti^3+^ (BT/rBT)	458.255/458.170	6920.090/15 538.830	1.541/1.221
Ti^4+^ (BT/rBT)	459.830/459.300	4229.967/1138.511	2.785/1.199
Ti^3+^ (BT/rBT)	463.945/463.488	2449.244/4327.734	2.060/1.749
Ti^4+^ (BT/rBT)	465.020/464.433	2240.286/4191.682	2.889/1.526
Lattice O (BT/rBT)	531.637/531.415	30 332.440/32 142.790	3.875/2.271
Absorbed O (BT/rBT)	529.444/529.375	4984.089/21 128.310	1.353/1.264

**Fig. 5:**
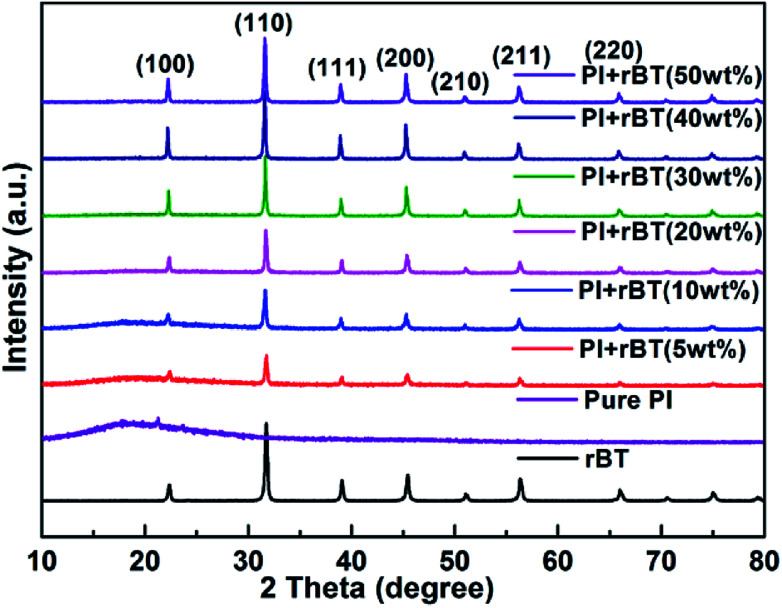
XRD patterns of the rBT/PI composites with different mass fractions of 0, 5, 10, 20, 30, 40 and 50 wt%.

Fig. 5 shows XRD patterns of the fabricated rBT/PI composite films. With increase in mass fractions of rBaTiO_3_ particles, all composite samples presented a typical polycrystalline perovskite structure of rBaTiO_3_ particles, characterized by (100), (110), (111), (200), (211) and (211) peaks as compared to rBaTiO_3_ particles.^52^ Moreover, the relative intensities of rBT diffraction peaks gradually increased with rBaTiO_3_ concentrations. The amorphous structure of pure PI was clearly observed by the broad peak at around 2*θ* = 18°, which was due to the accumulation of PI polymer chains. However, the XRD pattern of polyimide in rBT/PI composite was different from that of pure polyimide. The peak intensities of PI slowly decreased, which was attributed to the high content of rBaTiO_3_ fillers in the composites. This result suggested that the crystallinity of the pure PI was affected by rBT particles.

**Fig. 6:**
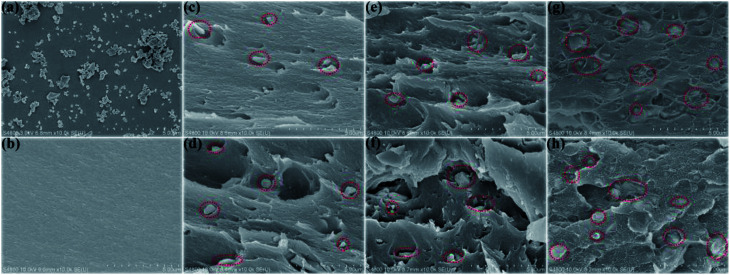
Scanning electron micrographs of the rBaTiO_3_ particles, (a) the fractured cross section of pure PI (b) and rBT/PI composites: (c) 5 wt% rBT; (d) 10 wt% rBT; (e) 20 wt% rBT; (f) 30 wt% rBT; (g) 40 wt% rBT; (h) 50 wt% rBT.

Fig. 6(a) shows the SEM images of the rBaTiO_3_ particles. The rBaTiO_3_ particles exhibit plate-like shape, whose particle sizes about 200–500 nm (length or width). The fractured cross-sections of pure PI and rBT/PI composites with different filler fractions are presented in Fig. 6(b)–(h). The fabricated rBT/PI composite films were approximately 7 μm to 14 μm in thickness. Under SEM observation, rBT particles showed no obvious aggregation and were homogeneously dispersed in the PI matrix even when the filler content was up to about 50 wt%. The homogeneous dispersion of the rBT particles contributed to the good dielectric properties of rBT/PI composites.^53^

**Fig. 7:**
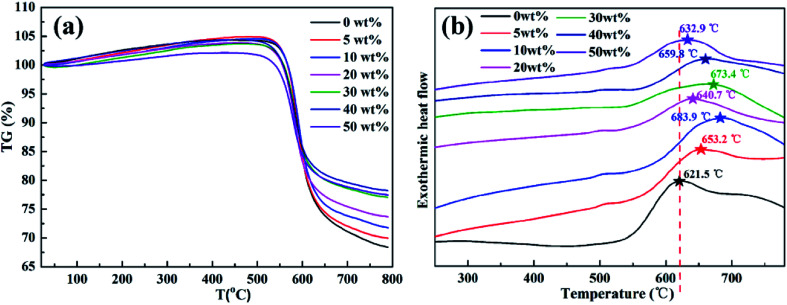
(a) TG and (b) DSC curves of pure PI and rBT/PI composites with different contents of fillers (0 wt%, 5 wt%, 10 wt%, 20 wt%, 30 wt%, 40 wt% and 50 wt%).

The thermal stability of pure PI and rBT/PI composites was investigated by TG and DSC, and the curves are shown in Fig. 7. In Fig. 7(a), the TG curves of the rBT/PI composites showed a similar trend as pure PI in the temperature range from 20 to 800 °C, indicating that the rBT powders had no influence on the degradation mechanism of PI matrix.^54^ Pure PI and rBT/PI composites started to degrade at 510 °C, which may be mainly due to the decomposition of the PI network.^55^ The values of weight loss rate of composites decreased with the increase in rBT loading, ranging from 31.6% (*x* = 0 wt%), 30.0% (*x* = 5 wt%), 28.2% (*x* = 10 wt%), 26.3% (*x* = 20 wt%), 22.9% (*x* = 30 wt%), 21.8% (*x* = 40 wt%), to 22.5% (*x* = 50 wt%). Overall, the rBT/PI composites had better thermal stability compared to pure PI, which was ascribed to the homogeneous distribution of rBT particles in the PI matrix.^56^ The rBT particles acted as barriers in the composites. Consequently, the volatile by-products formed during the pyrolysis could not escape.^57^ As shown in Fig. 7(b), the crystallization temperature of pure PI was about 621.5 °C. With increasing rBT contents, the crystallization temperature shifted to higher temperature, for example, 653.2 °C (*x* = 5 wt%), 683.9 °C (*x* = 10 wt%), 640.7 °C (*x* = 20 wt%), 673.4 °C (*x* = 30 wt%), 659.8 °C (*x* = 40 wt%), 632.9 °C (*x* = 50 wt%), respectively. The crystallization temperature of rBT/PI composite films rose with increasing rBT content, indicating that the crystallization process was promoted in the polymer matrix through the introduction of rBT powders.^58^

### 3.2. Dielectric properties of the rBT/PI composite films

**Fig. 8:**
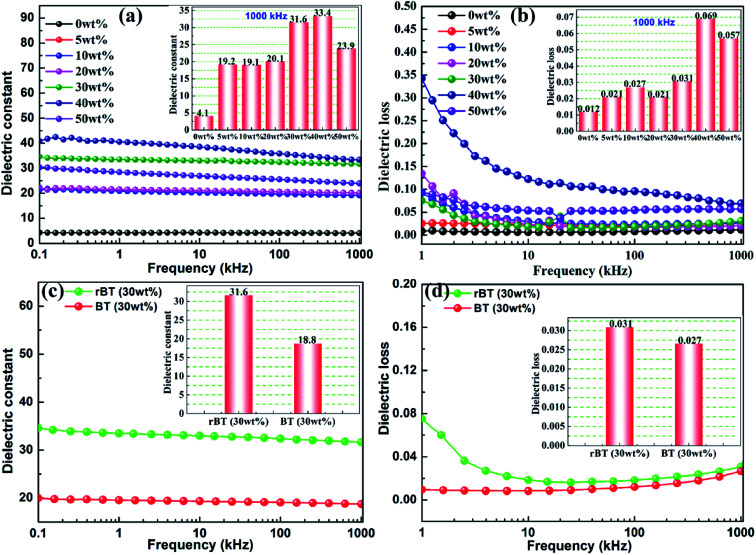
Frequency dependence of dielectric constant (a) and loss (b) of rBT/PI composites with different rBaTiO_3_ contents, and frequency dependence of dielectric constant (c) and (d) loss of rBT/PI (30 wt%) and BT/PI (30 wt%) composites. The insets are the dielectric constant and loss values corresponding to the fixed composition and measured frequency (1000 kHz).

The frequency dependence of dielectric constant and dielectric loss for rBT/PI composites with various mass fractions is illustrated in Fig. 8. The dielectric constant and dielectric loss of rBT/PI composites exhibited a decreasing trend with increase in frequency. This could be due to the response characteristics of different molecule groups and different chemical structures in different frequency ranges.^59^ Especially, the dielectric loss was relatively high in the low frequency range. The production of dielectric loss can be ascribed to the accumulation of many free charges at the internal interfaces between rBT and PI matrix. The interface polarization increases with the accumulation of free charges under the applied electric field.^60^ In the low frequency range, the charges have enough time to accumulate on the interfaces between rBT and PI matrix, which leads to high dielectric loss. In the high frequency range, the interfacial polarization cannot respond to the change in frequency, and thus, the dielectric constant and loss would decrease.^61^ In the insets of Fig. 8(a) and (b), the dielectric constant and dielectric loss of composite samples changed significantly with different rBT fillers. When the content of rBT increased up to 40 wt%, the dielectric constant reached its maximum of about 33.4 at 1000 kHz, which is considerably higher than that of pure PI (*ε*_r_ = 4.1). Subsequently, the dielectric constant sharply decreased to 23.9 with 50 wt% of rBT content. Correspondingly, the dielectric loss of samples was lower (tg *δ* < 0.031) when the rBT content was below 30 wt%. It was found that the rBT/PI composite with the loading of 30 wt% was the best candidate (*ε*_r_ = 31.6, tg *δ* = 0.031@1000 kHz) among all the tested composites.

The rBT/PI composite sample with 30 wt% was selected for comparison with the corresponding non-reduced composite filled with BaTiO_3_ particles. Dependence of dielectric constant and dielectric loss on frequencies for rBT/PI and BT/PI composites is shown in Fig. 8(c) and (d). The dielectric constant of rBT/PI composite film (*ε*_r_ = 31.6) was evidently higher than the BT/PI composite (*ε*_r_ = 18.8) at 1000 kHz, while maintaining a relatively lower loss (tg *δ* = 0.031) compared to that of BT/PI composite (tg *δ* = 0.027).

**Fig. 9:**
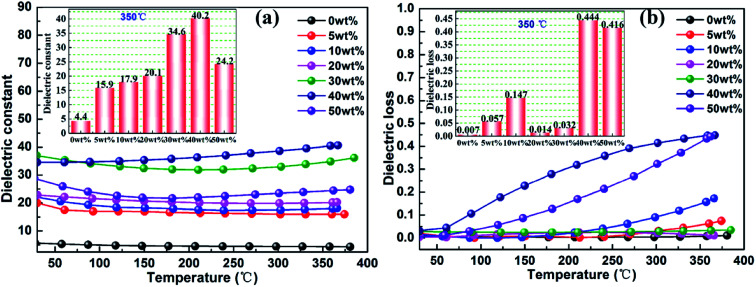
Temperature dependence of dielectric constant (a) and loss (b) of rBT/PI composites with different rBT concentrations, the insets are the curves of dielectric constant and loss *vs.* rBT concentration at 350 °C and 1000 kHz.

Fig. 9 shows the effect of temperature on the dielectric properties of rBT/PI composites. The dependence of dielectric constant and dielectric loss on temperature ranging from 25 to 370 °C at 1000 kHz was investigated. The dielectric constant of the rBT/PI composites showed no significant fluctuations over the entire temperature range. With increase in temperature, the dielectric constant increased slightly. This may be due to the high temperature resistance of PI. The dielectric loss of rBT/PI composites increased sharply with increase in temperature from 25 to 370 °C when rBT concentration was above 40 wt%. At lower content of rBT, the dielectric loss maintained a lower value over a wide temperature range (tg *δ* < 0.08). Generally, the statistical thermal motion of dipole and the charge distribution decide the dielectric properties of the composites.^62^ In Fig. 9(a) and (b), the dielectric constant of composites, measured at 350 °C, gradually increased with rBT content, and reached its maximum of about 40.2. Dielectric loss showed a lower value (<0.147) when rBT concentration was below 40 wt% than at high concentrations of rBT.

**Fig. 10:**
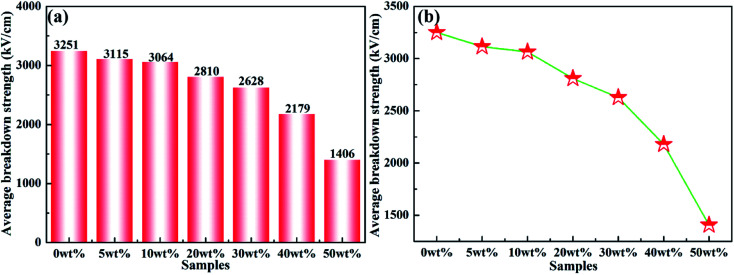
(a) and (b) Average breakdown strength of rBT/PI composites filled with different rBT contents.

Dielectric breakdown strength (*E*_b_) of composite materials is a crucial parameter to realize capacitor applications. Fig. 10(a) shows the average breakdown strength of rBT/PI composites with different rBT contents. The detailed *E*_b_ values at room temperature are listed in Table 2. Clearly, the *E*_b_ values gradually decreased with increasing rBT content, as shown in Fig. 10(b), ranging from 3251 kV cm^−1^ (*x* = 0 wt%), 3115 (*x* = 5 wt%), 3064 kV cm^−1^ (*x* = 10 wt%), 2810 kV cm^−1^ (*x* = 20 wt%), to 2628 kV cm^−1^ (*x* = 30 wt%), respectively. With further increase in rBT content, the *E*_b_ values reduced significantly from 2179 kV cm^−1^ (*x* = 40 wt%) to 1406 kV cm^−1^ (*x* = 50 wt%).

**Table 2:** Dielectric and energy storage properties of rBT/PI composite films

Samples	Dielectric constant/loss (1000 kHz)	Dielectric constant/loss (350 °C)	Average breakdown strength (kV cm^−1^)	Energy storage density (J cm^−3^)	Sample thickness (μm)
0 wt%	4.1/0.012	4.4/0.007	3251	1.9	7
5 wt%	19.2/0.021	15.9/0.057	3115	8.2	10
10 wt%	19.1/0.027	17.9/0.147	3064	7.9	7
20 wt%	20.1/0.021	20.1/0.014	2810	7.0	8
30 wt%	31.6/0.031	34.6/0.032	2628	9.7	14
40 wt%	33.4/0.031	40.2/0.444	2179	7.0	8
50 wt%	23.9/0.057	33.2/0.042	1406	2.1	14

**Fig. 11:**
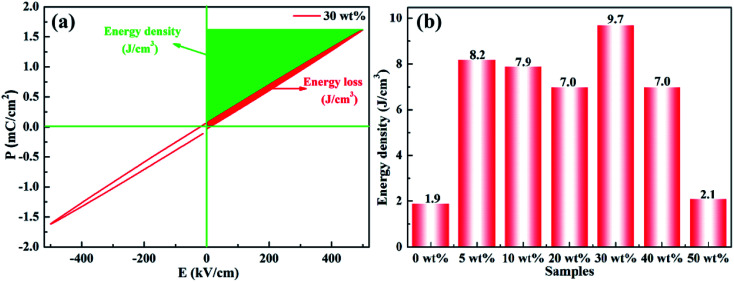
(a) Room temperature *P*–*E* loops of rBT/PI composites with 20 wt% rBT, (b) energy storage density of rBT/PI composites filled with different rBT contents.

**Fig. 12:**
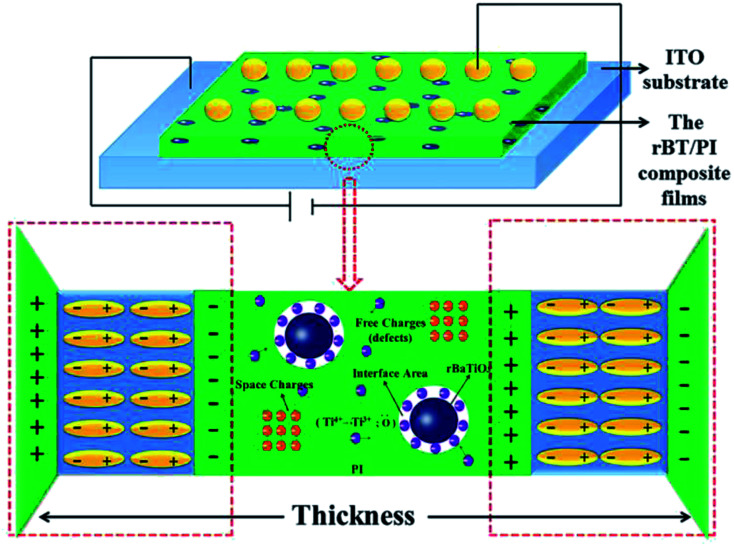
Schematic model of polarization mechanism for rBT/PI composite films.

The polarization–electric field (*P*–*E*) curve for a representative composite sample (*x* = 30 wt%) is presented in Fig. 11(a). It can be seen from the curve that at the electric field of 500 kV cm^−1^, the maximum polarization reached 1.616 μC cm^−2^, and the remanent polarization (*P*_r_) remained at a very low value, almost close to zero. A large energy-storage efficiency of about 90% (energy loss = 10%) was achieved in the composite film. *P*–*E* curves at higher electric fields (>500 kV cm^−1^) are not provided due to limitation of instruments. Similar trend was also observed in other composite samples (not shown here). According to the *P*–*E* loop, it was evident that the rBT/PI composite film possessed linear dielectric performance. Therefore, its energy storage density (*U*_e_) was calculated using the following formula:

2 *U*_e_ = 1/2*ε*_r_*ε*_0_*E*_b_^2^

where *ε*_r_ and *ε*_0_ are the relative dielectric constant and vacuum permittivity, respectively. *E*_b_ is the dielectric breakdown strength. The calculated results of energy storage density for different samples are shown in Fig. 12(b) and Table 2. Based on the overall consideration of *E*_b_ and *ε*_r_, a high energy density of 9.7 J cm^−3^ was achieved at 2628 kV cm^−1^ in rBT/PI composite filled with 30 wt% rBT particles. The energy density of the rBT/PI composites was enhanced by more than 400% over that of pure PI (1.9 J cm^−3^ at 3251 kV cm^−1^). Moreover, the energy density of rBT/PI composites was greater than the energy density of BOPP (1.2 J cm^−3^ at 6400 kV cm^−1^).^63,64^ Therefore, such marked enhancement of energy storage density (*U*_e_) was a result of introducing rBT particles in the polymer matrix to form the corresponding composite films. Moreover, another favorable property of rBT/PI composites was their ultrahigh energy storage efficiency. These results indicate that rBaTiO_3_ can effectively improve the dielectric constant and energy storage density of PI-based composites.

The above results can be explained according to the proposed polarization mechanism occurring in rBT/PI composites, as shown in Fig. 12. As discussed earlier, there are numerous defects inside the reduced BaTiO_3_, which are caused by lattice defects including a large number of oxygen vacancies and reduced Ti^3+^ ions. When rBT particles are introduced into PI to form composites, many space-charge polarization dipoles occur on the interface between rBT particles and PI due to a concentration gradient, which then contribute to the dielectric constant of composites.^65,66^ As shown in Fig. 8(a), a significant enhancement in dielectric constant was clearly observed at about 30 wt% rBT content. Moreover, the presence of these defects in rBT would facilitate the interface interaction between ceramic particles and polymer, which play an important role in dielectric loss and dielectric breakdown strength properties of composites.^67,68^ In this work, –COOH chains in PI matrix likely reacted with surface –OH groups of BT to form ester group. The existence of defects in rBT enabled the two phases to bond tightly to each other. These two factors were beneficial for the higher dielectric breakdown strength of rBT/PI composites at a certain content of rBT. However, the increase in rBT content not only enlarged the distance between rBT particles, but also extended the internal stress between the two phases. This may lead to the appearance of microcracking, and consequently lower the breakdown strength. Therefore, when the rBT content was increased up to 40 wt%, the dielectric breakdown strength of rBT/PI composite films decreased significantly.

## 4 Conclusions

In summary, reduced BaTiO_3_ (rBT) particles obtained by heating under reducing atmosphere were introduced into PI polymer matrix to form composite films by *in situ* polymerization method. It was found that the rBT with surface defects played an important role in enhancing dielectric and energy storage properties of PI-based composites. A significant increase in dielectric constant (*ε*_r_ = 31.6@1000 kHz) was realized for the composite sample with 30 wt% rBT, while maintaining a relatively low dielectric loss (tg *δ* = 0.031) and high breakdown strength (2628 kV cm^−1^). The measured energy density for the sample with 30 wt% rBT was as high as 9.7 J cm^−3^, which was much higher than that of pure PI (1.9 J cm^−3^). These results suggest that the rBT/PI composites could have potential applications in embedded capacitors.

## Conflicts of interest

There are no conflicts of interest to declare.

## Supplementary Material
